# How to Crack Pre-registration: Toward Transparent and Open Science

**DOI:** 10.3389/fpsyg.2018.01831

**Published:** 2018-09-26

**Authors:** Yuki Yamada

**Affiliations:** Faculty of Arts and Science, Kyushu University, Fukuoka, Japan

**Keywords:** QRP, misconduct in research, academic publishing, preregistration, open science

The reproducibility problem that exists in various academic fields has been discussed in recent years, and it has been revealed that scientists discreetly engage in several questionable research practices (QRPs). For example, the practice of *hypothesizing after the results are known* (HARKing) involves the reconstruction of hypotheses and stories after results have been obtained (Kerr, 1998) and thereby promotes the retrospective fabrication of favorable hypotheses (cf. Bem, 2004). P-hacking encompasses various untruthful manipulations for obtaining *p*-values less than 0.05 (Simmons et al., 2011). Such unethical practices dramatically increase the number of false positive findings and thereby encourage the intentional fabrication of evidence as the basis of scientific knowledge and theory, which leads to individual profits for researchers.

## Benefits of pre-registration

Pre-registration is a remedy for this problem that involves the submission of research papers for which experimental and analytical methods, including researchers' motivation and hypotheses, have been designed and described completely prior to the collection of actual data (similar to proposal papers). The date on which the research was registered is also recorded. The associated manuscript cannot be modified after research has been registered. In reviewed pre-registration, manuscripts are peer-reviewed prior to registration, and only manuscripts that successfully pass this stage are registered and will be published, regardless of whether the collected data support the registered hypothesis (resulting in publications known as registered reports). It has been repeatedly argued that pre-registration can be a powerful approach for addressing prevalent QRPs (Miguel et al., 2014; Munafò et al., 2017; Nosek et al., 2018). For example, pre-registration can prevent or suppress HARKing, p-hacking, and cherry picking since hypotheses and analytical methods have already been declared before experiments are performed. In cases involving reviewed pre-registration, publication is guaranteed at the registration stage, thereby preventing the occurrence of QRPs. A previous study reported that more than 30% of psychological researchers admitted to the involvement of QRPs (John et al., 2012). Since the object of such researchers who engage in QRPs may be to publish as many research papers as possible, pre-registration eliminates the necessity for such QRPs.

Furthermore, registered reports undergo an additional peer-review stage not present in the conventional publication process. Peer review is conducted both at the time of registration and after results have been reported. The reviewed pre-registration process is relatively laborious for researchers since it requires receiving a decision of acceptance from a journal editor on at least two separate occasions. Therefore, registered reports are considered to be authentic, and research results consistent with postulated hypotheses can achieve greater credibility and approval.

## Misuse of pre-registration

The preceding paragraphs provide a narrative about QRPs that can be effectively discouraged by pre-registration. However, a detailed examination of the current pre-registration system also reveals problems that this system cannot address. As mentioned, recognition of the value of pre-registration with respect to being able to confer reliability on research findings is becoming increasingly widespread. In terms of reputation management, researchers are motivated to improve their reputation regarding the credibility of their research (and themselves). A subset of researchers may attempt to misuse the pre-registration process to enhance their reputation even if their personality characteristics are not associated with readily engaging in data fabrication or falsification. Alternatively, certain situations may cause normal researchers to misuse this process on a momentary impulse (Schoenherr, 2015; Motyl et al., 2017). Their goals are to enhance the credibility of their research by pre-registering and to show the excellence of their hypothesis by presenting data that support that hypothesis.

There are methods for camouflaging a registered study as successful (van 't Veer and Giner-Sorolla, 2016). One such method is *selective reporting*, which is a type of data fabrication in which data that do not support the hypothesis are not reported (Goodman et al., 2016). Similarly, in the case of *infinite re-experimenting*, malicious researchers repeatedly perform the same experiment multiple times until the desired data to support the hypothesis are obtained and then report these data. Such QRPs cannot be completely prevented unless third parties can manage all of the data from experiments performed by researchers following registration. There is also a method that I call *overissuing*. Researchers who engage in overissuing pre-register a large number of experiments with extremely similar conditions and ultimately report only successful studies. This practice is difficult to discover by reviewers and editors who do not know a researcher's overall registration status; to date, this approach has not been explicitly identified as a QRP.

Another method is an approach that I call *pre-registering after the results are known* (PARKing). Researchers engaging in this practice complete an experiment (possibly with infinite re-experimentation) before pre-registering and write an introduction that conforms to their previously obtained results. Because such researchers apparently get attractive results and misrepresent those results as having been obtained under pre-registration, the research can readily acquire false credibility and impact. Rigorous initial peer-reviews that require revision of protocols may be able to reduce PARKing to some extent, but it is not effective if the malicious researchers involved engage in over issuing or target journals with poor peer-review practices. Furthermore, even if all unprocessed data are shared in a repository, the time stamps of uploaded data files can easily be forged or tampered with in various ways, such as by changing the system date for the operating system that is handling the data file. Therefore, there is currently no method for journals or reviewers to detect PARKing. Because many research resources would be required to implement the unethical methods described above, given the discarding of data that do not fit researchers' hypotheses, such methods can most easily be implemented by laboratories with abundant funds. If the aforementioned QRPs become rampant, their use could not only avoid decreases in false positives (which is a substantial advantage of pre-registration) but also accelerate the Matthew effect of rich people becoming richer (Merton, 1968).

It is easier to fabricate data and falsify results than to engage in *cracking* pre-registration; therefore, why should researchers attempt to crack pre-registration at all? The answer depends on the associated risk. Because data fabrication is a clear case of research misconduct and is subject to punishment, the risk associated with revelation is large. On the other hand, many of the cracking methods introduced here can be performed by simply extending general research practices. For example, suppose that a researcher conducted a paper-based questionnaire survey in the typical manner (without pre-registration) and had obtained significant results that supported his/her hypothesis and written a manuscript about this research. In this case, barriers to PARKing by using the introduction and method sections of the manuscript and subsequently publishing the full article appear to be low. Excel files for data aggregation can be recreated after pre-registration. If such cracking techniques have benefits that outweigh the difficulties and can be used with little risk, researchers who engage in these techniques will readily emerge.

## Beyond pre-registration

Therefore, pre-registration should not be overly trusted: it can easily be cracked. This paper introduces the idea that to prevent such cracking, registered research reports should not be completely believed just because “they were registered”; instead, several replications of the reported research with pre-registration should be performed. In addition, outsourcing experiments to multiple laboratories and agencies that do not share profitable interests with those of the registered researchers can be an effective means of preventing QRPs. If researchers outsourced experiments directly by themselves, some conflicts of interest could arise, so this process should be handled by journals who have received pre-registration protocols. In such cases, a journal would select an outsourcing partner for experimenting based on the protocol with the names of the original researchers being blinded. The candidate for outsourcing could either be selected by the journal in the same manner as reviewer selection or else crowdsourced. In either case, what matters is the precision of the experiment carried out, that this information is preserved for each candidate as a history, and that it can be used in the analysis or on subsequent request.

Preparing funds to implement this is a problem. It is to be hoped that financial support will be provided via various sources of funds based on the idea that such expenditure would help avoid the dissemination of numerous studies that involve the use of cracking approaches. Specifically as part of fraud countermeasure, universities or institutions to which the researchers belong could require them to underwrite this cost. Another way would be for the journal concerned to issue a coupon allowing the researchers entrusted with the experiments to use it as a resource for their academic activity. Indeed, this system has already been proposed for peer review (Gurwitz, 2017). Hopefully, national/international funding agencies should support and manage outsourcing replication efforts. It would also be effective for fostering a normative standard for high-quality research.

We should change the “positive results = win” mode of thinking that is pervasive throughout the scientific community. An important consideration is transparency. The conventional philosophy of pursuing positive results shrouds research in a fog. How, then, do we bring about such transparent practices? The first step may be to disseminate the pre-registration system to the utmost (i.e., make it mandatory). This will shift the value of pre-registration from an ethical device for distinguishing between ethical and unethical studies to that of research transparency that clearly divides theoretical and empirical work. If all the research is pre-registered, the ethics of that research is not governed by the pre-registration itself. Therefore, at this point, the cracking methods mentioned earlier in this article will lose efficacy.

The second step is research dividing, a successor model to pre-registration. Here I propose a new idea that theoretical and empirical elements no longer need to coexist in one paper. That is, someone can write a paper covering only theoretical elements, while someone else can write a paper focusing solely on empirical material. In the former, the theoretical validity and appropriate hypothesis formation are evaluated; in the latter, appropriate experimentation and analysis are assessed. Detailed discussion will be carried out by those who write a paper on a theoretical issue that advances the previous theory based on those results. Indeed, some idea journals are already in existence (e.g., *Medical Hypotheses* and the *Frontiers* journal's “Hypothesis and Theory”). Such a division of research will promote replication studies as being more natural and easier to conduct. Currently, the hurdle for reviewed pre-registration is too high for many researchers to conduct replication studies. However, for papers focusing solely on empirical material, it would be possible to conduct replications without pre-registration.

If this second step were achieved, the need for QRPs and research misconduct would be reduced. The “positive results reign supreme” attitude in the science community would be discarded because it would not be the yardstick by which researchers would evaluate. As long as the scientific publication system itself is transparent, reliable, and ethical, individual research would not need to be concerned with evaluation of such aspects. The best way to crack pre-registration is to abandon the fixed idea of the structure of scientific articles.

## Author contributions

The author confirms being the sole contributor of this work and has approved it for publication.

### Conflict of interest statement

The author declares that the research was conducted in the absence of any commercial or financial relationships that could be construed as a potential conflict of interest.
